# Unusual occurrence of orbital hemangiopericytoma in the zygomatic bone of an adolescent: a case report

**DOI:** 10.1186/s40662-018-0105-2

**Published:** 2018-05-13

**Authors:** Bahram Eshraghi, Hadi Ghadimi, Zohreh Nozarian

**Affiliations:** Eye Research Center, Farabi Eye Hospital, Tehran University of Medical Sciences, Qazvin Sq, Tehran, 1336616351 Iran

**Keywords:** Hemangiopericytoma, Orbital neoplasm, Solitary fibrous tumor, Zygomatic bone

## Abstract

**Background:**

Hemangiopericytoma and solitary fibrous tumor are considered related variants on the same spectrum and both may essentially be the same tumor. They are infrequently encountered in the orbital region while the zygomatic bone is an extremely rare location for these neoplasms to occur.

**Case presentation:**

A 14-year-old boy presented with complaint of deformity of left infraorbital area and a firm, regular mass in the region. Orbital CT scan revealed a well-defined round isodense intraosseous lesion in the lowermost portion of the lateral orbital wall (zygomatic bone), expanding the bone and protruding anteriorly and medially. MRI showed the mass to be heterogenous and strongly enhancing with contrast medium. Inferior transconjunctival orbitotomy was performed and the mass was removed. The histopathologic examination and immunohistochemistry staining results (positive for CD34, CD31 and smooth muscle actin, but negative for CD99, S100, B-cell lymphoma 2 (bcl-2) and desmin) confirmed the diagnosis of hemangiopericytoma. The postoperative course was uneventful, with no evidence of recurrence after 5 years follow up.

**Conclusions:**

This case represents the second hemangiopericytoma reported in the zygomatic bone. Although extremely rare, hemangiopericytoma/solitary fibrous tumor might be considered in the differential diagnosis of intraosseous lesions of the orbital and zygomatic region.

## Background

Vascular tumors of the bone are uncommon, accounting for 1–2% of bone tumors [1]. Hemangiopericytoma (HPC) is an infrequent vascular neoplasm that has a propensity for soft tissues and rarely involves bony skeleton [2], constituting 4.7% of primary vascular bone tumors [3]. Occurrence of hemangiopericytoma in the zygomatic bone is extremely rare, with only one previous report in the literature [4]. We present an adolescent with HPC of zygoma who underwent successful surgical treatment.

## Case presentation

**Fig. 1:**
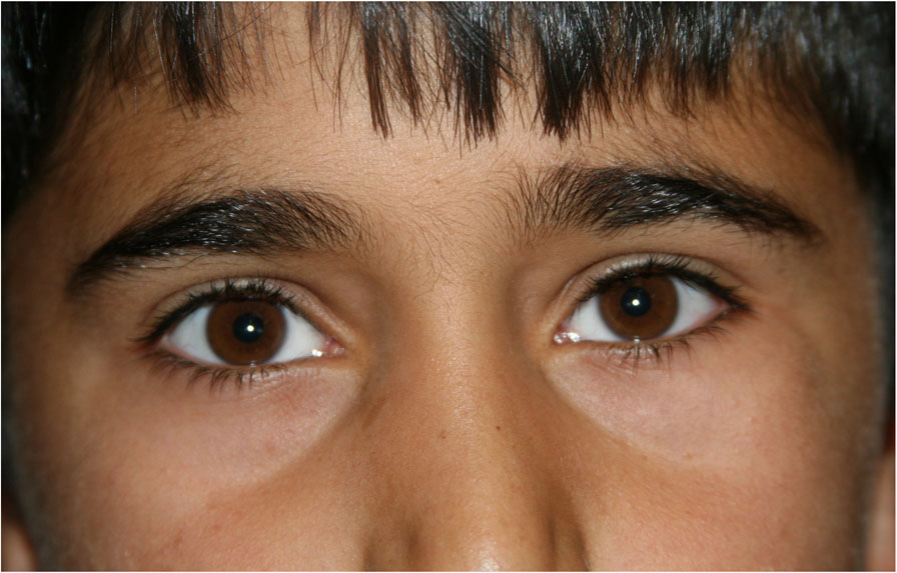
Preoperative facial photograph. Frontal view of the patient before operation, showing fullness of left inferolateral orbital and periorbital region with slightly upward displacement of the lateral canthus

A 14-year-old boy was referred with complaint of deformity of left infraorbital region. There was fullness in the inferolateral orbital and periorbital areas (Fig. 1) and a firm, regular mass was palpable in the region. The patient had felt the bulging for almost a year. No limitation of ocular motility was observed, but lateral canthus was slightly displaced superiorly compared to the other side. The mass was neither painful nor tender and the overlying skin appeared normal. Past history was unremarkable and the patient denied history of any trauma.

**Fig. 2:**
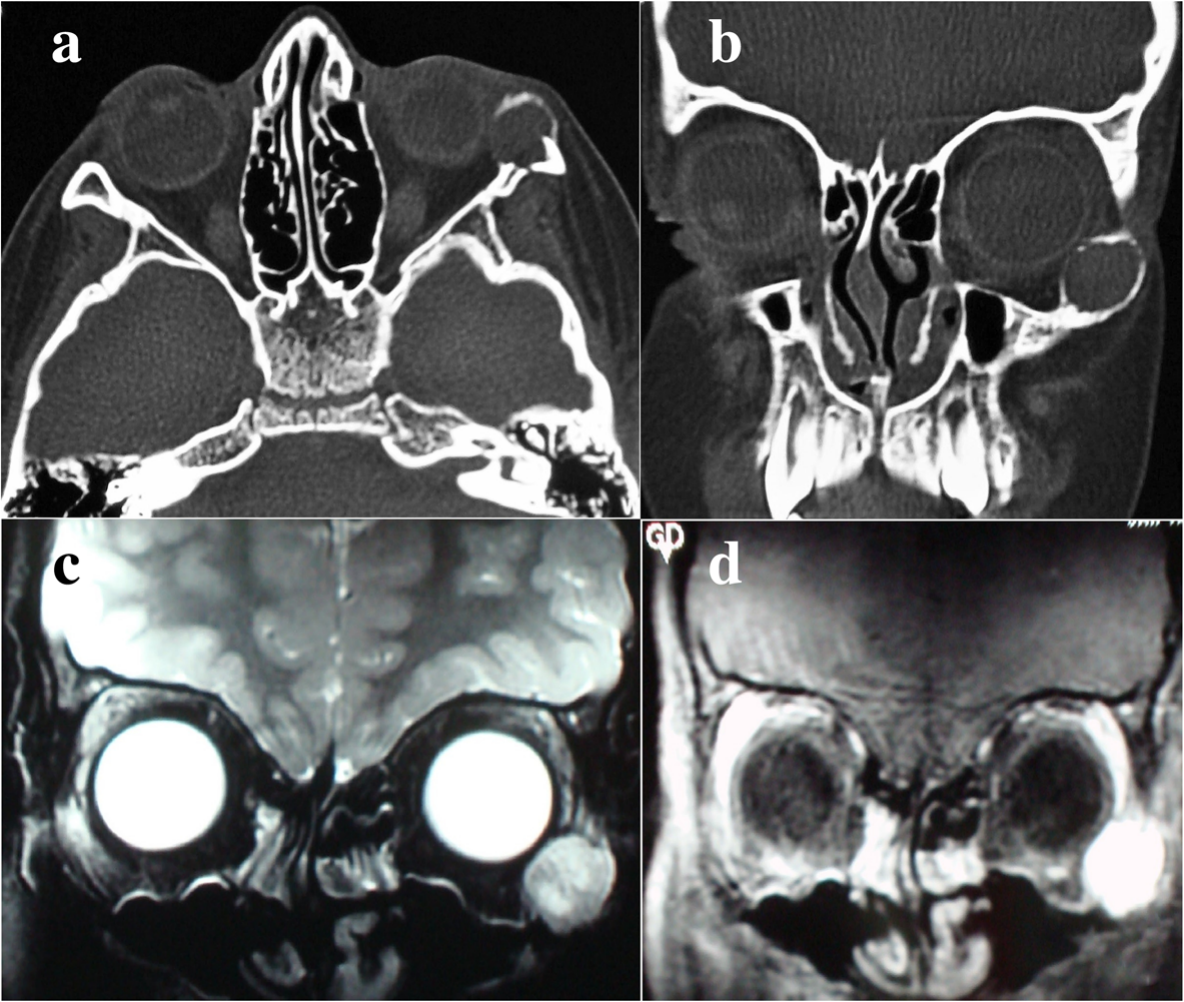
Preoperative imaging. CT scan reveals an isodense intraosseous lesion in the zygomatic bone in axial (**a**) and coronal sections (**b**). In orbital MRI, the mass appears heterogenous in T2-weighted images (**c**) and shows remarkable contrast enhancement after gadolinium injection (**d**)

Orbital CT scan revealed a well-defined round isodense intraosseous lesion in the lowermost portion of the lateral orbital wall, expanding the bone and protruding anteriorly and medially (Fig. 2a and b). MRI showed the mass to be heterogenous and strongly enhancing with contrast medium (Fig. 2c and d). Based on available information, the clinical suspicion of a vascular lesion was aroused. Under general anesthesia, inferior transconjunctival orbitotomy was performed and the mass was exposed. The bony roof of the lesion was removed, piecemeal excision of its contents was carried out by curettage and hemostasis was achieved with the aid of Surgicel (Ethicon, Somerville, NJ). Intralesional triamcinolone acetonide was injected at the termination of surgery before suture closure of the conjunctiva.

**Fig. 3:**
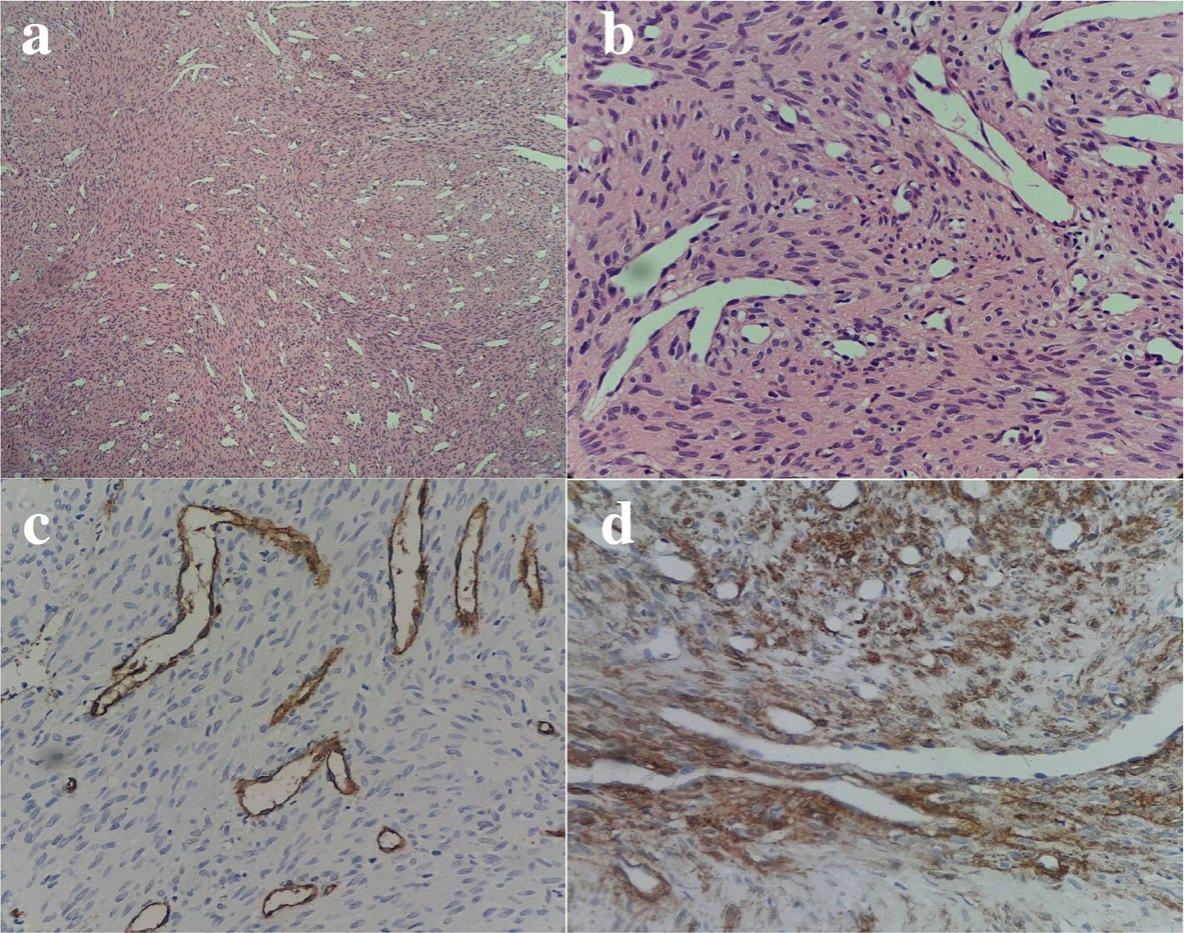
Pathologic findings. Histopathologically (**a** and **b**), the tumor is composed of several slit-like vascular channels with occasional stag-horn appearing structures, surrounded by numerous spindle cells (H&E stain, low and high magnification, respectively). Immunohistochemistry shows positive staining for CD31 and CD34 in endothelial cells of blood vessels (**c**) and also positive staining for smooth muscle actin (SMA) in spindle cells (**d**)

**Fig. 4:**
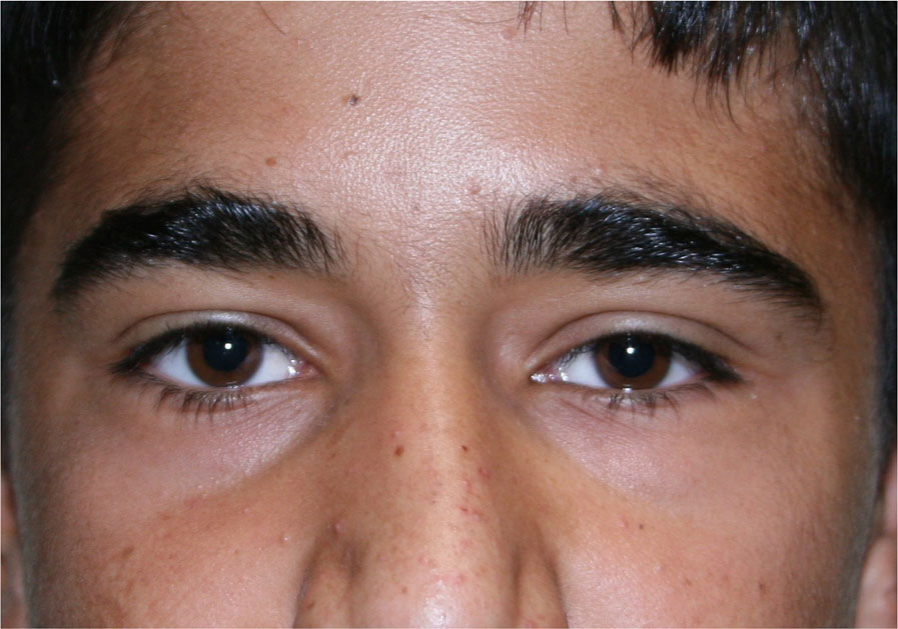
Postoperative facial photograph. Frontal view of the patient 5 years after the surgery, showing complete resolution of inferolateral orbital fullness

**Fig. 5:**
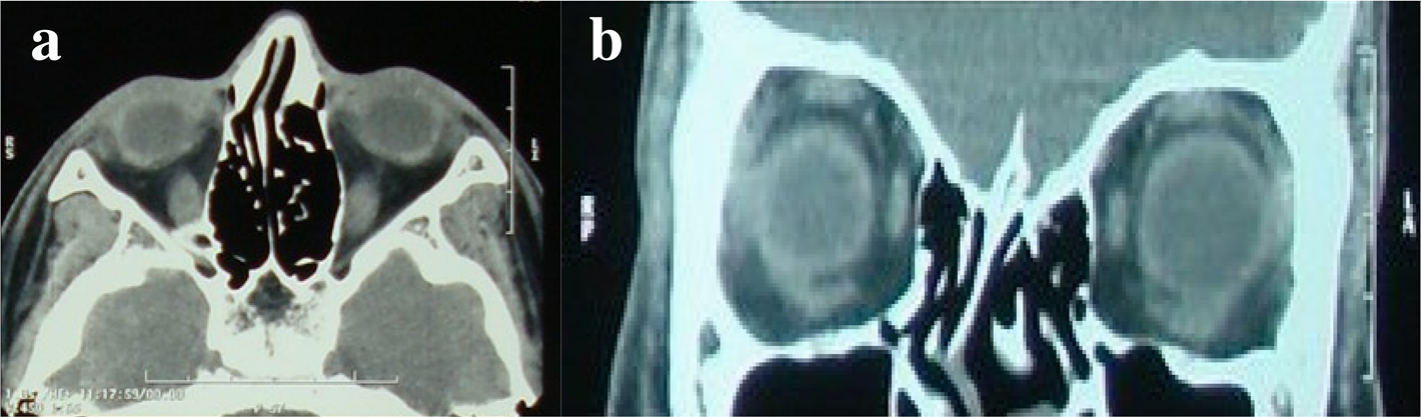
Postoperative imaging. Orbital CT scan reveals mild secondary hyperostosis in the previous location of the mass, with reasonable healing and no remnant of the original tumor (**a**: axial section, **b**: coronal section)

Histopathologic examination revealed the mass to be composed of several slit-like vascular channels, with occasional stag-horn appearing vessels, surrounded by elongated bland-looking spindle cells (Fig. 3a and b). Occasional mitotic figures (1 per 20 high power fields) were seen. As the specimen was received as fragmented pieces, exact evaluation of surgical margins was not possible. However, in some of the larger pieces evaluated, surrounding soft tissue and margins showed no tumor involvement. Immunohistochemistry (IHC) staining results were positive for CD34 and CD31 in endothelial cells (Fig. 3c) and for smooth muscle actin (SMA) in spindle cells (Fig. 3d), but negative for CD99, S100, B-cell lymphoma 2 (bcl-2) and desmin, suggesting the presence of HPC. The postoperative course was uneventful, with the patient doing well without clinical or radiologic signs of recurrence after 5 years follow up (Figs. 4 and 5a, b).

## Discussion and conclusions

HPC is a rare vascular soft-tissue tumor that can develop anywhere in the body that has blood vessels, most notably in lower extremities, pelvic fossa, retroperitoneum and nasopharynx [5]. HPC rarely involves the orbit [6] and constitutes only 1.57% of orbital tumors [7]. Likewise, bones are uncommonly involved in HPC, which accounts for 4.7% of bone tumors [2, 3]. Zygomatic bone involvement by intraosseous malformations is rare and HPC of the zygoma has only been reported once [4]. This case represents the second reported instance of intraosseous HPC of the zygoma. In the case reported by Asrani et al. [4], a diagnosis of HPC of the zygoma was made although IHC results were not presented, and the patient was followed for 2.5 years after surgery.

HPC is primarily a tumor of adults, with the median age of 45 years upon presentation, and without predilection to either sex [5]. The clinical and radiological features of HPC are not specific nor characteristic and diagnosis is made by histopathological examination [8]. Patients usually present with a painless mass that grows slowly [8]. The typical appearance of HPC on the CT scan is that of a well-circumscribed mass lesion with occasional calcifications. MRI may better show the heterogeneity of tumor components with areas of low and high signal intensity on T2-weighted images representing calcifications and thrombosis/old hematoma within the mass, respectively [9].

The microscopic appearance of HPC is remarkable for branching or “staghorn” thin-walled blood vessels with densely packed spindle cells oriented randomly [10]. Solitary fibrous tumor (SFT) is a closely related entity that shows substantial overlap of clinical and morphologic characteristics with HPC, and many authors recently considered HPC and SFT as variants belonging to the same spectrum [11]. The most prominent differences in histopathological features include variable cellularity, foci of dense collagenization and strong CD34 reactivity in SFT compared with trivial variability in cellularity, minimal collagenization and focal CD34 staining in HPC [12–14]. However, HPC and SFT are indistinguishable in many instances and most of the entities under the ill-defined term of HPC have progressively escaped from this category, leaving some cases that are now recognized as cellular or malignant forms of SFT rather than HPC [15]. Most periocular tumors identified as HPC are nowadays recognized as SFT and both may essentially be the same tumor, despite some morphologic and immunologic variations [16]. The most recent World Health Organization (WHO) classification of soft tissue tumors has rendered the term HPC obsolete and categorizes all such tumors within the SFT group [17, 18], except for those presenting in the CNS that continue to be regarded as different entities [19].

Immunohistochemically, HPCs show reactivity for CD34, vimentin, and SMA, but are mostly negative for desmin and S-100 protein [10, 11, 15]. Despite the potentially malignant/aggressive nature of HPCs, attempts at prediction of clinical behavior of the tumor based on pathological characteristics have largely failed, which makes HPC an unpredictable neoplasm [8, 20]. Nevertheless, features like tumor size (diameter greater than 6.5 cm), mitotic activity (more than 4 mitoses per 10 high power fields), hypercellularity, anaplasia, necrosis and hemorrhage have been reported as indicators of malignant behavior [5, 8].

The treatment of choice for HPC is complete en-bloc surgical excision whenever possible, because these tumors are most often circumscribed or enclosed by a pseudocapsule [7, 11]. Completeness of surgical resection positively affects survival, while incomplete excision (piece-meal resection) or incisional biopsy are associated with local spread, recurrence and metastasis [7, 11]. However, as in our case, complete tumor resection can occasionally be challenging due to its friable and highly-vascular nature [21]. Local recurrence rate is 33% [20] and it typically occurs years later in those who had piece-meal tumor resection [5, 8]. Therefore, long term follow up is essential to detect possible recurrence or metastasis. Therapeutic options available for recurrent HPC include further attempts at surgical excision, orbital exenteration, excision combined with adjuvant radiotherapy, and chemotherapy [6, 21].

Recent advances have been made in the understanding of the genetic basis of SFT/HPC [22]. SFTs are consistently associated with NAB2-STAT6 gene fusions [23, 24]. NAB2 (NGFI-A binding protein 2) is a transcriptional repressor of early growth response genes (EGR1). STAT6 (signal transducer and activator of transcription 6) is a transcriptional activator that has a role in interleukin 4 signaling [25]. NAB2 and STAT6 are both located closely on the chromosomal band 12q13. Their fusion results in the production of a chimeric protein that induces cellular proliferation through the activation of EGR1 [23, 24]. It has been shown that nuclear expression of the carboxy terminal part of STAT6 was highly specific and sensitive for SFT, allowing SFT to be distinguished from its histological mimics [26].

In conclusion, an extremely unusual site of involvement (zygomatic bone) by a rare tumor (HPC/SFT) is reported and the relevant literature is reviewed. Complete surgical excision provides the most effective treatment, although postoperative follow up is required for a long period of time.

## Abbreviations

EGR1: Early growth response
HPC: Hemangiopericytoma
IHC: Immunohistochemistry
NAB2: NGFI-A binding protein 2
SFT: Solitary fibrous tumor
SMA: Smooth muscle actin
STAT6: Signal transducer and activator of transcription 6
